# Applying a participatory system dynamics approach to childhood overweight and obesity in the local context: reflections from the LIKE project

**DOI:** 10.1186/s12961-025-01345-5

**Published:** 2025-05-26

**Authors:** Angie Luna Pinzon, Karien Stronks, Arnoud Verhoeff, David Vaandrager, Karen den Hertog, Wilma Waterlander

**Affiliations:** 1 Amsterdam UMC Location University of Amsterdam, Department of Public and Occupational Health, Amsterdam Public Health Research Institute, Meibergdreef 9, Amsterdam, The Netherlands; 2 Public Health Service (GGD), City of Amsterdam, Sarphati Amsterdam, Nieuwe Achtergracht 100, 1018 WT Amsterdam, The Netherlands; 3 Department of Sociology, University of Amsterdam, 1018WV Amsterdam, The Netherlands; 4 Department of Healthy Living, City of Amsterdam, Public Health Amsterdam, 1018WT Amsterdam, The Netherlands

**Keywords:** System dynamics, Public health, Obesity prevention, Case study, Evaluation, Systems thinking

## Abstract

**Background:**

Methods based in system dynamics (SD) have gained prominence within public health research in recent years. SD is grounded in theory and explains how central principles, such as adaptation, dynamics and emergence can be used to understand and/or change complex systems. To date, few examples exist where this theory has been applied consistently in a prevention approach in a local context. This study aimed to reflect upon the application of theoretical SD principles in context of the Lifestyle Innovations Based on Youth Knowledge and Experience (LIKE) project.

**Methods:**

A multi-methods qualitative evaluation was conducted using the LIKE project, situated in Amsterdam, the Netherlands, as a case study. LIKE applied a participatory system dynamics approach for obesity prevention in youth, throughout the project during a time period of 6 years (2017–2023). Data collection included document reviews, a Ripple Effects Mapping workshop, and semi-structured interviews with involved stakeholders, followed by in-depth reflective analysis.

**Results:**

We identify three key lessons combining theory and practice: (1) theory: interdependency programme and context; lesson: avoid becoming overly focused on achieving a complete understanding of the system related to the topic under study (for example, obesity). Instead, ensure sufficient attention is given to comprehending the dynamics of the local context, including existing initiatives and policy processes; (2) theory: dynamic and adaptive character; lesson: while the ability to encompass real-world dynamics is a foundational strength of system dynamics theory, its practical application can be constrained by more static elements, such as budget planning, and the need for clearly defined roles and responsibilities; and (3) theory: strong governance; lesson: SD projects require strong governance including strategic planning and enduring commitment, but in the absence of clear milestones or measurable impact on the short term.

**Conclusions:**

Applying SD principles in practice requires a collective shift in thinking and working for all parties involved. Challenges in particular relate to the many uncertainties that arise whereby everything continues to change over time, including the focus of the system under study; relevant stakeholders; and momentum for change. This necessitates strategies different from our accustomed linear research working practices, shifting instead towards more iterative approaches that accommodate complexity and uncertainty.

## Background

The recent (2022) World Health Organization European Region Obesity Report provides a clear insight into the severity and complexity of (childhood) overweight and obesity and how it results from the interaction of multiple factors operating at various system levels [1, 2]. The application of systems thinking – an approach centred on understanding and addressing complex problems – has gained prominence within public health in recent years [3, 4].

Systems thinking is not a unified or singular field; rather, it encompasses a diverse and evolving set of methods drawn from disciplines, such as general systems theory, complexity science, and organizational learning; whereby methods have been applied and further developed in the study of natural, mechanical, and social systems [5, 6]. Among these, system dynamics has emerged as particularly influential in public health. Originally developed by Jay Forrester (1961), early works included industrial dynamics, urban dynamics, world dynamics and the national economic model [7]. From here, system dynamics introduced key concepts, such as feedback loops, time delays, and non-linear relationships, which helped explain how complex behaviours and patterns can emerge over time through adaptive responses and interactions between system elements [8–11].

Ideally, public health programmes grounded in the theoretical principles of system dynamics acknowledge this rich intellectual history and adopt its broader line of thinking, rather than limiting themselves to isolated methods – such as causal loop diagrams or social network analysis – applied only during specific phases of a project [5]. Rather, projects should encompass the system dynamics perspective across all phases of the programme – design, implementation, and evaluation [9, 11]; and integrate key characteristics of SD.

There are few examples of public health programmes in the literature applying a SD perspective from start to finish [12]. One example is the Lifestyle Innovations On the basis of Youth Knowledge and Experience (LIKE) project. LIKE combined SD and participatory action research in a 6-year transdisciplinary collaboration between academia, policy, and practice with the aim of creating healthier living environments for adolescents (age 10 to 14) [13]. LIKE aimed to divert from the traditional linear intervention development-implementation-evaluation approach and instead embraced SD principles by developing a programme that was: (a) inextricably linked to the context in which it was implemented, aiming to change this context while being influenced by it; and (b) developing an approach that is adaptive; whereby the programme instigates changes within the targeted system, and responds to these changes and recognizes that the system itself undergoes continuous transformations, influenced not only by the programme but also by external factors [14–16]. It is important to clarify that, in using the term “system,” we adopt a constructivist systems thinking perspective – understanding a system as a conceptual tool to make sense of complex, messy situations. As Rutter and Koorts describe, “the notion of a ‘system’ is not necessarily a fixed, bounded entity, but rather a way of understanding interconnections and patterns”. This stands in contrast to interpretations of systems as reified or objective entities, such as a hospital system or school system [11, 17].

Recently, we published the findings of the LIKE programme, in terms of the implementation of the action programme, how it targeted different system levels and how it changed over time [18].

Alongside the output of the LIKE action programme, we also obtained important learnings from conducting the LIKE project as a whole. Specifically, in applying a SD approach – with interdependency between the programme and context, dynamic and adaptive properties, and transdisciplinary stakeholder involvement –in the practicalities of a local context. Studies adopting a SD approach have touched upon these practicalities [19, 20]; however, a recent review [21] noted limited guidance on how SD principles can be applied in practice and how to best manage the unpredictable nature of SD approaches [22–24].

In this paper, we therefore aim to complement the findings from the LIKE action programme output paper [18] by reflecting on: (a) how central theoretical principles of SD were applied in practice; (b) the associated challenges encountered and; (c) lessons learned, in the local context of a city district in Amsterdam, the Netherlands.

## Methods

### The LIKE project: an overview

LIKE was embedded within the Amsterdam Healthy Weight Approach (AHWA), a local-government-led whole systems approach aiming to reduce childhood overweight and obesity in Amsterdam [36]. In LIKE, we worked closely with adolescents, families and community stakeholders to develop, implement, and evaluate an adaptive action programme. Herein the LIKE project followed a six-stage cyclic theory of change process: (1) needs assessment; (2) mapping the pre-existing system; (3) identifying leverage points; (4) developing an action programme; (5) monitoring action programme adaptation; and (6) evaluating programme impact (9). This paper covers all the six project stages; and Table 1 provides details on the theoretical underpinnings, objectives, and activities for each stage. Here, we specifically focus on the LIKE project as a whole; rather than separate actions or interventions that were developed along the way, which are published separately in the above mentioned output paper [18] and individual results papers [37–39].

**Table 1:** Summary of the six stages of the LIKE project, including the theoretical underpinning and objectives for each stage

	Phases 1 and 2: conducting a needs assessment and mapping the pre-existing system		Phases 3 and 4: identifying leverage points and developing an action programme		Phase 5 + 6: monitoring action programme adaptation and capturing programme impact	
Theoretical underpinning and objectives	SD approaches initially focus on developing an understanding of the system, referred to as the pre-existing system [9]. This understanding generally includes the perspectives of relevant actors, acquired through multiple methods [25, 26]. Additionally, it also includes an understanding of both the system’s structure and functioning [27].		Once a system understanding is acquired, the next step involves identifying where to intervene, referred to as leverage points [8, 10, 25]. Various frameworks exist that can be used to locate leverage points [28, 29] and which distinguish the different levels related to both the structure and functioning of the system. From these leverage points, an action programme is developed and implemented in an iterative process of adaptation and reflection.		Phase five monitors action programme implementation. This includes assessing how the programme adapts over time as new insights emerge, and identifying the contextual factors that influence its implementation. It is important to monitor how the wider system adapts and reacts to the action programme and use this information to modify the programme accordingly [20, 30]. There is a strong emphasis on learning while doing, enabling necessary adjustments to be made to achieve the desired outputs and outcomes in phase six.	
Theoretical elements to consider	• SD foundational work, soft systems thinking, critical systems thinking [31]: System boundaries; Context sensitivity; Multiple perspectives of actors • Community-based system dynamics[32]: Participatory; multiple and mixed methods	• Classical systems thinking [7, 10]: System elements; non-linear relationships; and feedback loops; Underlying system dynamics; Systems-based analysis: differentiate between system’s structure and functioning	• Transition theory, tipping points [33]: Examine disruption potential for systems change • Leverage points [10]: Leverage points at different system levels	• Evaluation[14, 34]: Define theories of change; Distinguish between action function and action form • Community-based system dynamics[32]: Engage relevant stakeholders	• Complex systems thinking, evaluation theory [26, 34, 35]: Adaptation in response to new or changing conditions; Real-time feedback; Develop, test, retest, and adjust programme	Uncertainty regarding processes and outcomes; Intended and unintended outcomes

### Study design

Details about the overall design of the LIKE evaluation are available elsewhere [9]. For this paper, we conducted retrospective qualitative analysis of document reviews (for example, minutes), a Ripple Effects Mapping (REM) workshop, and semi-structured interviews (*n* = 12) with LIKE project members. The resulting data were triangulated to provide a comprehensive reflection on implementing a SD project in a real-world setting. This study was approved by the institutional medical ethics committee of Amsterdam UMC, Location VUMC (2018.234).

### Data collection

#### Document reviews

We extracted information about the project’s development and implementation over time from minutes of various LIKE project meetings, including:

- *LIKE steering committee* (*n* = 5 members; with approved notes from *n* = 27 meetings);
- *LIKE consortium* (around *n* = 30 members; with approved notes from *n* = 18 meetings);
- *LIKE evaluation team* (*n* = 4 members; with approved notes from *n* = 9 meetings).

#### Ripple effects mapping workshop

In March 2022, a REM workshop with LIKE consortium members (*n*=10) was conducted with the specific aim of capturing the effects of the LIKE project as a whole. This REM workshop was held separately from a REM workshop covering the LIKE action programme as described in the LIKE output paper [18].

REM, a qualitative participatory method suitable for evaluating systems approaches [40, 41], creates a mind map illustrating programme activities, their consequences (ripples), and resulting outcomes. The two-hour workshop, following a standard facilitator script [42], featured a hybrid format and focused on two central questions: (1) what insights has LIKE provided? These include substantive (for example, understanding of the system), procedural (for example, managing collaboration), and methodological insights; and (2) how have you applied these insights in your work?

#### Interviews

We conducted twelve interviews between October 2022 and June 2023 to deepen the findings from the REM maps. Participants were purposefully selected based on their involvement in various LIKE project stages, and included academic researchers (*n* = 3), AHWA professionals (*n* = 3), LIKE consortium leaders (*n* = 3), and professionals from the Amsterdam local government (*n* = 3). The interviews, following a semi-structured guide, lasted approximately 60 min and occurred either in-person or online.

### Data analysis

All interviews were transcribed verbatim, and we employed inductive thematic analysis using Microsoft Word to identify emerging themes within previously mentioned three key theoretical aspects of SD programmes. Subsequently, we conducted a more in-depth reflective analysis of the programme development and implementation by integrating insights from the reviewed documents and the REM mind map, which complemented the emerging themes from the interview data. Finally, the main findings were presented to the consortium leaders for final input and feedback.

## Results

### The interdependency between the programme and the local context in which it is applied

#### How did we shape this principle?

LIKE aimed to align the local context and SD perspective by co-creating the programme with adolescents, families, community stakeholders and local policy makers in terms of: (a) understanding the system; and (b) developing actions. An overview of all relevant activities is provided in Tables 1 and 2 and includes: developing causal loop diagrams (CLDs) based on the scientific literature [43]; PAR research with adolescents [37]; Group Model Building (GMB) [39]; construction of an overarching systems map and leverage points for change [27]; and Social Network Analysis (SNA).

**Table 2:** Delivery of the LIKE project across six stages

	Phases 1 and 2: Conducting a needs assessment and mapping the pre-existing system	Phases 3 & 4: Identifying leverage points and developing an action programme	Phase 5 + 6: Monitoring action programme adaptation and capturing programme impact
Delivery in LIKE	A mixed-methods needs assessment (2018–2021) captured three perspectives on the pre-existing system driving obesity-related behaviours in adolescents in Amsterdam: 1) Academic researchers constructed causal loop diagrams (CLDs) based on scientific literature[43] 2) Adolescents developed CLDs through participatory action research groups [38] 3) Group model building workshops with community stakeholders (for example, parents and teachers)[39] and interviews with healthcare professionals were conducted[44, 45]. Data integration resulted in a CLD containing 121 factors and 31 feedback loops. Systems-based analysis revealed six subsystems related to adolescent interactions with the food environment; physical activity environment; online environment; wider socioeconomic environment; healthcare and the transition from childhood to adolescence [27]. Social network analysis (SNA) was conducted to examine changes in the network regarding stakeholders’ collaborations to improve adolescents’ health. Two snapshots of this network were taken (Anselma et al., in preparation). Lastly, action mapping documented all relevant actions in the LIKE target area.	To arrive at the LIKE action programme, three approaches were followed. First, adolescents developed ideas through a participatory action research process [38]. Second, local stakeholders developed ideas through group model building workshops (Waterlander et al., in preparation). Adolescents and stakeholders mostly developed actions which targeted the structural levels of the system. Last, the LIKE consortium employed the Intervention Level Framework [28]to identify leverage points, and predominantly focused on developing actions which targeted the functional system levels[15]. The LIKE action programme had a total of 22 actions that were implemented. These targeted the following system levels: paradigm (*n* = 0), goals (*n* = 2), system structure (*n* = 4), feedback and delays (*n* = 3) and structural elements (*n* = 13)[18].	The LIKE evaluation team (ALP, WW, KS) kept track of information about action characteristics (including setting, action form, and specified theory of change) and action development progress (including facilitators, barriers, system level and stakeholders involvement) in an action monitoring registry. Plenary meetings were also organized by the evaluation team (between 2020 and 2022) with LIKE members to keep the monitoring registry up to date. Additionally, information concerning the outputs and outcomes of the action programme was collected through a Ripple Effects Mapping workshop and by conducting interviews with LIKE members[18].

Furthermore, we documented ongoing activities in the community relevant to health-related behaviours of adolescents in an action registry and observational data were collected by being physically present in the target neighbourhoods and engagement in community initiatives. Finally, we aimed to align actions with the local policy context, by our collaboration with the AHWA.

#### Reflective analysis

It is natural for academic projects to start with an analysis of the problem under study (obesity). Accordingly, we spent a lot of time understanding the local system dynamics of childhood obesity; a process that took 2–3 years, resulting in a comprehensive multi-perspective CLD [27]. While useful, we also learned that it is equally, or possibly even more important, to focus on understanding the context in terms of: who relevant stakeholders are; how (local) government is organized; what is happening both on and beneath the surface; where possible momentum for change can be found; and how to stay on top of this information as it changes over time. For example, we started with developing an action registry at the start of the project, but underestimated how fast this context changes:

“Yes, but I also think that we as researchers never have any idea how much is already being done. And what we saw in Amsterdam East is that we were increasingly at home there, I was there more and more, I cycled there more and more. Man, just walk into a community centre there and see what’s organized. You can’t see the forest for the trees.” (P5)

Relatedly, we learned that many actions are not directly visible, such as getting a topic on the political agenda or the formation of a new belief that obesity prevention is a collective responsibility. To capture these relatively hidden activities and capitalize on their momentum, a solid understanding of the context you’re working in is required. System analysis, for example using the Intervention Level Framework [28] or Action Scales Model [29]; while monitoring ongoing activities is a useful starting point in capturing this information.

A related reflection is on the application of SNA, which is a frequently used method to map stakeholder networks. While this method has merits in providing a graphic and measurable picture of the local context (numbers of stakeholders and relationships between them); it is not particularly suitable when trying to “work” with this context. The question “who relevant stakeholders are” changes over time and depends on the focus of the programme. Initially, our focus was concentrated on the public health domain and, through SNA, we had a relatively solid idea which organizations were working on this topic in the community. But, as the programme developed, we learned that the council also had relevant programmes for citizen participation or poverty reduction, and none of those stakeholders were in the map:

“It’s true that you can never involve all stakeholders beforehand because you don’t know which activities you’re going to do, but perhaps, in this case, in the beginning, especially in Amsterdam East, we should have paid much more attention to it.” (P11).

### The adaptive character of the programme

#### How did we shape this principle?

The adaptive nature of the LIKE programme was reflected in the choice to structure the project around a six-stage cyclic process. We visualized this process using the ENCOMPASS framework [9]; which functioned as a broad theory of change, guiding how the project aimed to facilitate systems changes (Table 1).

During the initial phases of needs assessment and understanding the pre-existing system, research questions were formulated exploratively, without a pre-specified focus on a particular part of the system. For example: “What factors and processes in the pre-existing system in Amsterdam East shape unhealthy behaviours?” and “For those parts of the system that will be addressed by the action programme: what does the pre-existing look like in terms of relevant stakeholders, power relations, and ongoing policies and activities?” This allowed the programme to evolve over time. For the same reason, we applied broad system boundaries.

Similarly, the developed action ideas were not fixed, but instead were based on a theory of change and adapted over time, based on ongoing input from researchers (scientific literature) and stakeholders (local context). Accordingly, the involved stakeholders varied, depending on the focus of the action idea. A crucial factor for feasibility was an unallocated budget accessible to the action-groups. This budget did not impose substantive requirements on the focus of the action, only that the action-groups indicated the targeted system parts using the Intervention Level Framework, considered a theory of change, and planned how to evaluate the action.

#### Reflective analysis

An open approach is essential to allow a SD programme to evolve over time and to develop actions tailored to the local context. However, this approach carries the risk of fragmentation. In hindsight, we underestimated the importance of defining a clear focus and making strategic choices. For example, narrowing the system boundaries after establishing an initial system understanding could have strengthened the coherence. In Amsterdam, there was significant momentum to work on the food environment and formalize youth participation in policy development. Instead of developing actions across all subsystems, focusing on these two subsystems might have achieved deeper system-level actions.

The adaptive character of our approach and responsiveness to real-world dynamics, also posed challenges in maintaining stakeholder engagement. Given the programme’s broad focus, we initially involved a wide range of stakeholders. However, as the action programme evolved, only specific stakeholders were needed at each stage. This raises important questions: what happens to the other stakeholders? Conversely, how do you ensure that stakeholders have the time to seize opportunities at a specific moment in time, like during national elections? This process requires specific guidance, which will be discussed further in the next section on governance.

A final reflection concerns the timing of sharing findings among stakeholders. We observed that researchers faced challenges in sharing scientific insights early on, before completing a thorough analysis. Researchers hesitated because they were unsure whether their findings were “true” and would hold up under peer review. Similarly, local government partners were hesitant to involve their staff members, because they were already overcommitted and were unsure which expertize was required at each stage.

### Governance of a system dynamics approach

#### How did we shape this principle?

Organizing the governance was a continuous effort of the LIKE project, and like everything else, adapted over time based on the changing context. At the project’s outset, two governance levels were installed: (1) a steering committee with one or two senior representatives from each of the involved sectors (*n* = 5); and (2) the full consortium (approximately *n* = 30 members). At the start of LIKE’s second year, a third governance level – positioned between the existing levels – was added in the form of a Management Board consisting of all project leaders and supervisors (*n* = 15). The Management Board focused on the process decisions of LIKE, while the full consortium concentrated more on the (scientific) content.

To reflect on and enhance the governance, we organized a full-day workshop with a professional coach midway through the LIKE project. This workshop coincided with the project’s transition from the initial two phases – system insight – to the subsequent two phases – action. It aimed to clarify roles and responsibilities during these two phases and our joint expectations in the collaboration.

Using the workshop’s outcomes, we again re-structured the project governance by installing transdisciplinary action-groups (see also the previous section). To oversee these action-groups and their progress, we scheduled general meetings every 6 weeks, where all action-groups reported back. These meetings replaced the management board meetings. A project coordinator was newly appointed to manage this process.

#### Reflective analysis

Investing in governance with all three sectors involved (academia, policy, practice) was essential for fostering trust and understanding each other’s way of working. This worked particularly well at the “highest” governance level, the steering committee:

“So, from those different (steering committee members) perspectives, we can have a very open conversation about it, and I respect what my colleagues say, and that’s mutual. (This is) what I eventually find wonderful about this journey, and what I consider one of the greatest lessons learned in this collaboration” (P8)

Extending team cohesion to the wider consortium proved challenging despite regular meetings. We substantially underestimated the time needed to understand the workings of the involved sectors at a level required to implement a SD approach successfully. From the start, ambiguity surrounded the roles and responsibilities of those involved in LIKE, particularly when tasks fell outside people’s job descriptions. As a result, most individuals only engaged in activities related to their specific expertize and lacked oversight of the whole project.

This ambiguity became particularly challenging around halfway through the project, where the focus shifted from understanding the system to developing actions. While academic researchers were more in the lead during the first phase, AHWA and municipal stakeholders were expected to take the lead in the second phase. However, this shift never really occurred, despite the aforementioned workshop around this theme; and we noticed that people were generally involved based on their topical expertize (for example, ethnic minorities or physical activity); rather than on cross-cutting skills such as guiding transitions or political engagement. This reflection illustrates the difficulty in navigating the balance between involving stakeholders embedded in the context and making the project dynamic. The dynamic nature poses challenges in predefining individual roles, given the uncertainty surrounding the project’s focus. As individuals involved in the project come from different organizations, supervising them is challenging because the person overseeing the whole lacks direct authority. In retrospect, we should have invested much more time and energy in this aspect of governance:

“I also think that as consortium leaders, throughout the entire process, you need to focus much more on managing the process. And you should be constantly vigilant about it. Where are we now? Which parties are involved? Does it still align? Are the roles and responsibilities clear? Do we need to steer in that direction? I believe that as consortium leaders in this case, you need to be continually very attentive, yes, more alert than we were, I think. And you need to be more actively steering.” (P11)

This type of governance is challenging because it requires equal partnership among science, policy, and practice, and oversight and leadership qualities across: (a) understanding and applying SD principles; (b) overseeing all ongoing activities and relevant stakeholders; and (c) applying an optimal approach to action navigation. In the LIKE project, these tasks were divided among different individuals, hindering synchronization.

## Discussion

SD has a solid theoretical foundation, but applying these principles in practice in a local context is not straightforward and requires a collective shift in thinking and working for all involved parties. Challenges particularly relate to the many uncertainties that arise during such projects whereby everything continues to change over time, including the focus of the system under study; the involved stakeholders; and the momentum for change.

From the project’s start, we were aware that a participatory SD approach would demand a new way of looking at the delivery of a public health programme. However, what this delivery would entail in practice was something that we learned along the way. We experienced that implementing a participatory SD approach requires a continuous navigation by all stakeholders involved, guided by the system boundaries and the project’s overall aim. In this process, we identified a number of distinct dilemmas; including: (1) applying a dynamic and adaptive approach versus making strategic choices within stricter system boundaries to generate greater impact; (2) involving relevant stakeholders without knowing the exact focus of the initiative; and (3) responding adequately to momentum or leverage points with involvement of the necessary subject-matter expertize.

The findings from this study add to the growing literature on factors influencing the implementation of SD approaches [22–24, 46, 47]. These studies generally describe the implementation process of SD using broad theoretical principles, such securing shared language and ownership among stakeholders [19]. While these principles make sense on paper, we learned their application is not straightforward, presents specific challenges, and can even be contradictory. For example, sharing academic findings early with wider stakeholders is important for developing shared ownership and involving the context. However, this also means sharing unvalidated information and using evidence in policy before scientific peer-review. Similarly, there might be strong political momentum to focus on a specific part of the system (that is, there is leverage), but the open character of the approach results in widespread activities, with no one ready to seize on this momentum. This involves making boundary judgments, and it is essential to engage in ongoing reflection on their ethical and political dimensions. While system dynamics offers valuable tools for understanding system behaviour, it has certain limitations in explicitly addressing these normative aspects. Other systems approaches – such as Soft Systems Methodology and Critical Systems Heuristics – provide alternative frameworks for examining such values or power dynamics [31]. By highlighting these dilemmas in our study, we hope to inspire future projects to anticipate such challenges.

Many observed challenges stem from governance. As discussed, trying to transform a system while simultaneously conducting a research project is a challenging combination. This demands a focused approach to governance and clear delineation of roles and responsibilities throughout the project. Furthermore, embracing a systems approach requires a collective shift in thinking and working for all involved parties [48]. Members from academia, policy, and practice involved in LIKE had to develop the skills of thinking and working within a systems perspective; and team members acknowledged the challenges they faced in applying principles of systems thinking to research (for example, how do we measure impact?) and daily work practices (for example, I recognize the complexity, but what do I do on Monday morning?) [49]. This relates to the tensions described by Ison, describing a conflict between systemic thinking (which values fluidity, feedback, and emergence) and systematic practice (which relies on fixed plans, roles, and structures) [6].

Our study reveals that successful application of participatory SD approaches in a local context requires specific conditions. Some of these conditions were met in our project. For instance, the grant provider did not mandate us to specify exact implementation activities, allowing for a dynamic approach. However, other conditions were not met, posing challenges within the project’s timeframe. Examples include the need for budgetary flexibility at the municipal level to involve relevant stakeholders in project activities, and having a good understanding of which stakeholders to involve in each stage. Nevertheless, at the start of the project, there were no examples of what applying a SD approach means and what potential challenges look like. In the meantime, some literature has become available on this topic, potentially helping future projects [12]. Reflecting on our journey, we set out to develop a project following the broad path of systems thinking – embracing complexity and systemic change. However, the practicalities of working in academic and governmental contexts, make it easy to divert to the narrower path of structured implementation. As Gadsby and Wilding (2024) observe, “The narrow path often feels safer, but it can distance us from the transformative potential of systemic thinking” [5].

## Strengths and limitations

This study is among the first to reflect on applying a participatory SD approach in a local policy context. To date, only three studies using this type of approach from start to finish have been identified in literature [12]. Consequently, a significant portion of our effort was dedicated to developing theory and methods while simultaneously trying to instigate systems changes and involve all relevant actors. With the rapid expansion of literature on this topic, future studies can focus more efficiently on generating systems changes, especially considering the availability of logic frameworks for adopting a SD approach. This advancement implies that some of the dilemmas, such as the interdependency between programme and context and governance structures, that we encountered and detailed in this study can be more proactively addressed.

## Conclusions

Applying a participatory SD approach in a local context demands an unconventional strategy, necessitating conditions different from our accustomed research working practices. Key principles, such as the interdependency between the implementation process and context, and the dynamic character of the approach, are crucial for success but also pose specific dilemmas during implementation, evaluation and governance. Embracing a SD approach necessitates a collective shift in thinking and working for all involved parties. We identify three key lessons: (1) interdependency programme and context: the tendency in these types of projects is to focus on what needs to be done content-wise to solve the problem (obesity), but equally important is having a clear understanding of the working of the local context, as that determines success; (2) dynamic and adaptive character: being “dynamic” is within the name of system dynamics and therefore seems logical to do, but application its practical application can be constrained by more static elements such as budget planning, and the need for clearly defined roles and responsibilities; and (3) governance: our study underscores that achieving meaningful systems changes and successfully implementing projects on the basis of such principles require strategic planning, effort, and enduring commitment. By the time everyone understands how to navigate complexities in systems transformations, a regular project (even lasting 6 years) is already completed. Our findings highlight the need for a long-term perspective and persistence when applying SD in public health, whereby we anticipate that future projects can learn from our experiences.

## Data Availability

No datasets were generated or analysed during the current study.

## Abbreviations

AHWP: Amsterdam Healthy Weight Programme
CLD: Causal loop diagram
LIKE: The Lifestyle Innovations Based on Youth Knowledge and Experience project
REM: Ripple effects mapping
SD: System dynamics
